# Management of the smaller twin with impending compromise in twin pregnancies complicated by selective fetal growth restriction: a questionnaire-based study of clinical practice patterns

**DOI:** 10.1186/s12884-023-05616-3

**Published:** 2023-05-12

**Authors:** So-hee Kim, Young Mi Jung, Chan-Wook Park, Joong Shin Park, Jong Kwan Jun, Mi Hye Park, Han Sung Hwang, Seung Mi Lee

**Affiliations:** 1 Department of Obstetrics and Gynecology, Seoul National University College of Medicine, 101 Daehak-ro, Jongno-gu, Seoul, 03080 Korea; 2 Department of Obstetrics and Gynecology, Cheju Halla General Hospital, Jeju, Korea; 3 Department of Obstetrics and Gynecology, Guro Hospital, College of Medicine, Korea University, Seoul, South Korea; 4 Department of Obstetrics and Gynecology, Ewha Womans University, Seoul, Korea; 5 Department of Obstetrics and Gynecology, Research Institute of Medical Science, Konkuk University School of Medicine, Seoul, Korea; 6 Department of Obstetrics and Gynecology, Konkuk University School of Medicine, 120-1 Neungdong-Ro (Hwayang dong), Gwangjin-Gu, Seoul, 05030 Korea; 7 Innovative Medical Technology Research Institute, Seoul National University Hospital, Seoul, South Korea; 8 Department of Obstetrics and Gynecology, Seoul National University Hospital, Seoul, South Korea

**Keywords:** Multifetal pregnancy, Selective fetal growth restriction, Viability, Intact survival, Timing of delivery

## Abstract

**Background:**

In twin pregnancies complicated by selective fetal growth restriction (sFGR), if the smaller twin is in the state of impending intra-uterine death (IUD), immediate delivery will reduce the risk of IUD of the smaller twin while exposing the larger twin to iatrogenic preterm birth (PTB). Therefore, the management options would either be to maintain pregnancy for the maturation of the larger twin despite the risk of IUD of the smaller twin or immediate delivery to prevent IUD of the smaller twin. However, the optimal gestational age of management transition from maintaining pregnancy to immediate delivery has not been established. The objective of this study was to evaluate the physician’s perspective on the optimal timing of immediate delivery in twin pregnancies complicated by sFGR.

**Methods:**

An online cross-sectional survey was performed with obstetricians and gynecologists (OBGYN) in South Korea. The questionnaire asked the following: (1) whether participants would maintain or immediately deliver a twin pregnancy complicated by sFGR with signs of impending IUD of the smaller twin; (2) the optimal gestational age of management transition from maintaining pregnancy to immediate delivery in a twin pregnancy with impending IUD of the smaller twin; and (3) the limit of viability and intact survival in general preterm neonates.

**Results:**

A total of 156 OBGYN answered the questionnaires. In a clinical scenario of dichorionic (DC) twin pregnancy complicated by sFGR with signs of impending IUD of the smaller twin, 57.1% of the participants answered that they would immediately deliver the twin pregnancy. However, 90.4% answered that they would immediately deliver the pregnancy in the same scenario for monochorionic (MC) twin pregnancy. The participants designated 30 weeks for DC twin and 28 weeks for MC twin pregnancies as the optimal gestational age of management transition from maintaining pregnancy to immediate delivery. The participants regarded 24 weeks as the limit of viability and 30 weeks as the limit of intact survival in general preterm neonates. The optimal gestational age of management transition for DC twin pregnancy was correlated with the limit of intact survival in general preterm neonates (p < 0.001), but not with the limit of viability. However, the optimal gestational age of management transition for MC twin pregnancy was associated with both the limit of intact survival (p = 0.012) and viability with marginal significance (p = 0.062).

**Conclusions:**

Participants preferred to immediately deliver twin pregnancies complicated by sFGR with impending IUD of the smaller twin at the limit of intact survival (30 weeks) for DC twin pregnancies and at the midway between the limit of intact survival and viability (28 weeks) for MC twin pregnancies. More research is needed to establish guidelines regarding the optimal delivery timing for twin pregnancies complicated by sFGR.

**Supplementary Information:**

The online version contains supplementary material available at 10.1186/s12884-023-05616-3.

## Background

Selective fetal growth restriction (sFGR) is defined as the estimated fetal weight of one twin < 10th centile plus intertwin weight discordance > 25% in a twin pregnancy [1]. In previous reports, the incidence of sFGR was similar between monochorionic (MC) and dichorionic (DC) twin pregnancies [2], but the neurological morbidity and other complications such as fetal death [3] was higher in affected MC twins due to shared placenta circulation [4, 5]. As most twins are delivered simultaneously, the decision for delivering or maintaining the pregnancy can affect both twins. In DC twin pregnancies, sFGR should be monitored similarly to growth-restricted singletons, and the optimal timing of delivery should be determined based on a risk-benefit assessment and the wishes of twin’s parents [1]. The options could either be to maintain the pregnancy for the maturation of larger twin with the risk of IUD of smaller twin or immediate delivery to prevent IUD of smaller twin. In MC twin pregnancies, the IUD of smaller twin can result in IUD or neurological damage in larger twin [6] because of the presence of inter-twin anastomosis. Therefore, if there is real risk of impending IUD of smaller twin in MC twins, the management options could be conservative management followed by the sacrifice of smaller twin (either by selective termination of smaller twin or observation of IUD of smaller twin after selective laser coagulation to prevent damage to larger twin) or immediate delivery to prevent IUD of smaller twin [7, 8] that may leads larger twin’s IUD or neurologic damage simultaneously due to hypoxic brain injury [6]. Therefore, in both DC or MC twin pregnancies with impending IUD of smaller twin, the clinical decision would either be to sacrifice the smaller twin or immediate delivery, and the decision between these two options is influenced by gestational age. Because MC twin pregnancy may have the greater impact on the larger twin in the situation of IUD of smaller twin than DC twin pregnancy, physicians are expected to decide immediate delivery in earlier weeks, but the optimal gestational age of management transition from sacrificing the smaller twin to immediate delivery has not been established. In addition, the twinning rate and the rate of PTB of twin pregnancy was increased compared with the past [9, 10].

Therefore, to improve the outcomes of twin pregnancies complicated by sFGR, it is essential to establish the optimal timing of immediate delivery. The main aim of this study was to evaluate the physician’s views on the optimal gestational age of management transition from maintaining pregnancy to immediate delivery in twin pregnancies complicated by sFGR with signs of impending IUD of smaller twin.

## Methods

### Study design

Between March 2020 and May 2020, the Korean Society of Ultrasound in Obstetrics and Gynecology (KSUOG) sent an electronic survey questionnaire to their full members and KSUOG web site registered members regarding their perspective on the optimal timing of immediate delivery in twin pregnancies complicated by sFGR for DC or MC twin pregnancies. The members of KSUOG consisted of physicians that were either obstetricians and gynecologists [OBGYN] board members or doctors in resident training for obstetrics and gynecology. The study was approved by the Institutional Review Board of the ethics committee of the Seoul National University Hospital (IRB No. 1908-201-1060).

### Questionnaire

In the electronic questionnaire, participants were asked questions regarding their baseline characteristics including their gender, age, main specialty, number of years of experience in obstetrics and gynecology, number of sFGR cases evaluated and delivered per year by themselves, level of their working hospital, and existence of neonatal intensive care unit (NICU) in their hospital. Then, they were asked about the management of twin pregnancies complicated by sFGR. In the questionnaire, sFGR was defined as the estimated fetal weight of one twin < 10th centile plus intertwin weight discordance > 25% in a twin pregnancy [1], and this definition was given to participants at the beginning part of the questionnaire. The clinical scenario in question was a twin pregnancy complicated by sFGR with signs of impending IUD of the smaller twin in DC or MC twin pregnancies. The questions were asked as follows: (1) In a given situation, what would you decide between immediate delivery for preventing IUD of a smaller twin or pregnancy prolongation for lowering the risk of PTB for larger twin?; (2) In your opinion, when is the optimal gestational age of management transition from maintaining pregnancy to immediate delivery in a twin pregnancy with impending IUD of the smaller twin?; and (3) For general preterm neonates, when fetuses can achieve the limit of viability and intact survival in gestational age?. In the questionnaire, intact survival was defined as neonatal survival without neurologic abnormality [11], and this definition was given to participants in the question. The questionnaire gathered quantitative and qualitative data from numerical, categorical, and multiple-choice questions. The total response rate was calculated based on the number of KSUOG members who received the electronic questionnaire and responded the questionnaires completely. The primary outcomes of this study were the optimal gestational age of management transition from maintaining pregnancy to immediate delivery in DC or MC twin pregnancies complicated by sFGR and its correlation with viability and intact survival.

### Survey method and statistical analysis

All data were analyzed using SPSS version 23.0 (IBM Inc., Armonk, NY, USA). The continuous variables were presented as median and interquartile range (IQR) and compared using Student’s *t*-test (Mann–Whitney U test). The categorical variables were presented as numbers and percentages and compared using the chi-square test (Fisher’s exact test). The correlation between optimal delivery timing and viability or intact survival was analyzed by nonparametric correlation analysis using Spearman’s rho coefficient. A p-value of < 0.05 was considered statistically significant.

For the study, the sample size calculation was performed to determine how many survey participants would be needed to check the difference of tendency of when would the participants decide to deliver in the situations of impending death of smaller twin complicated by sFGR in both case of DC and MC twin. We anticipated there would be about 10% differences in participants answers, between MC twin situation and DC twin situations and around 20% of response rate. We thought the participants would make life-saving choices, but this tendency would be more pronounced in MC twin. The calculation was done under 80% power and 5% of type I error, we determined that this study would need 153 of survey participants.

## Results

### Participants’ characteristics

A KSUOG sent questionnaires to 845 members. Among them, 156 KSUOG members answered the questionnaire, and calculated response rate is about 18.5%. The characteristics of participants are summarized in Table 1. Of the 156 KSUOG members, 65% of the participants were OBGYN board members, and more than half of these board members were maternal-fetal medicine specialists. The number of years of experience as OBGYN doctors was relatively equally distributed between 5 and 25 years. Most participants were working at a tertiary center (n = 110, 70.5%). About half of the participants evaluated and delivered more than 20 twin pregnancies per year.

**Table 1:** Baseline characteristics of the study population

Characteristics	Values, n (%)
Gender	
Male	42 (26.9%)
Female	114 (73.1%)
Age, years (n)	
20s	7 (4.5%)
30s	87 (55.8%)
40s	34 (21.8%)
50s	25 (16.0%)
60s	3 (1.9%)
Above 70s	0 (0.0%)
Profession	
Still in training	56 (35.9%)
OBGYN specialist	100 (65.1%)
Subspecialties	
Maternal-fetal medicine	82 (52.6%)
Other main practice	74 (47.4%)
Years practicing as a specialist	
Still in training	58 (37.2%)
< 5 years	28 (17.9%)
5–15 years	26 (16.7%)
16–25 years	33 (21.2%)
< 25 years	11 (7.1%)
Level of working hospital	
Primary care clinic	8 (5.1%)
Hospital	13 (8.3%)
General hospital	25 (16.0%)
Tertiary hospital	110 (70.5%)

Abbreviations: OBGYN, obstetricians and gynecologists

### Timing of delivery in situations of impending death of the compromised smaller twin

Tables 2, 3 and 4 shows the response of participants in situations of twin pregnancies complicated by sFGR with signs of impending IUD of the smaller twin.

**Table 2:** Choice of participants in situations of impending death of the compromised smaller twin in selective fetal growth restriction

	In DC twin	In MC twin
Immediate delivery to save smaller twin	89 (57.1%)	141 (90.4%)
Prolongation of pregnancy to reduce the risk of PTB of larger twin	67 (42.9%)	15 (9.6%)

Abbreviations: DC, dichorionic; MC, monochorionic; PTB, Preterm birth

**Table 3:** The physician’s perspective on the optimal delivery timing for impending compromise of the smaller twin in selective fetal growth restriction

	DC	MC	p-value
Optimal delivery timing in term of gestational age at delivery (weeks)	30 (23–35)	28 (23–34)	< 0.001

Data are presented as proportion (%) or median (Interquartile range)

Abbreviations: DC, dichorionic; MC, monochorionic

**Table 4:** The physician’s perspective on limits of viability or intact survival in preterm neonates

	GA (weeks)	EFW (g)
Viability	24 (22–28)	500 (250–1500)
Intact survival*	30 (23–35)	1000 (400–1700)

Data are presented as proportion (%) or median (Interquartile range)

GA, Gestational age; EFW, Estimated fetal weight

*Intact survival was defined as neonatal survival without neurologic abnormality

**Fig. 1:**
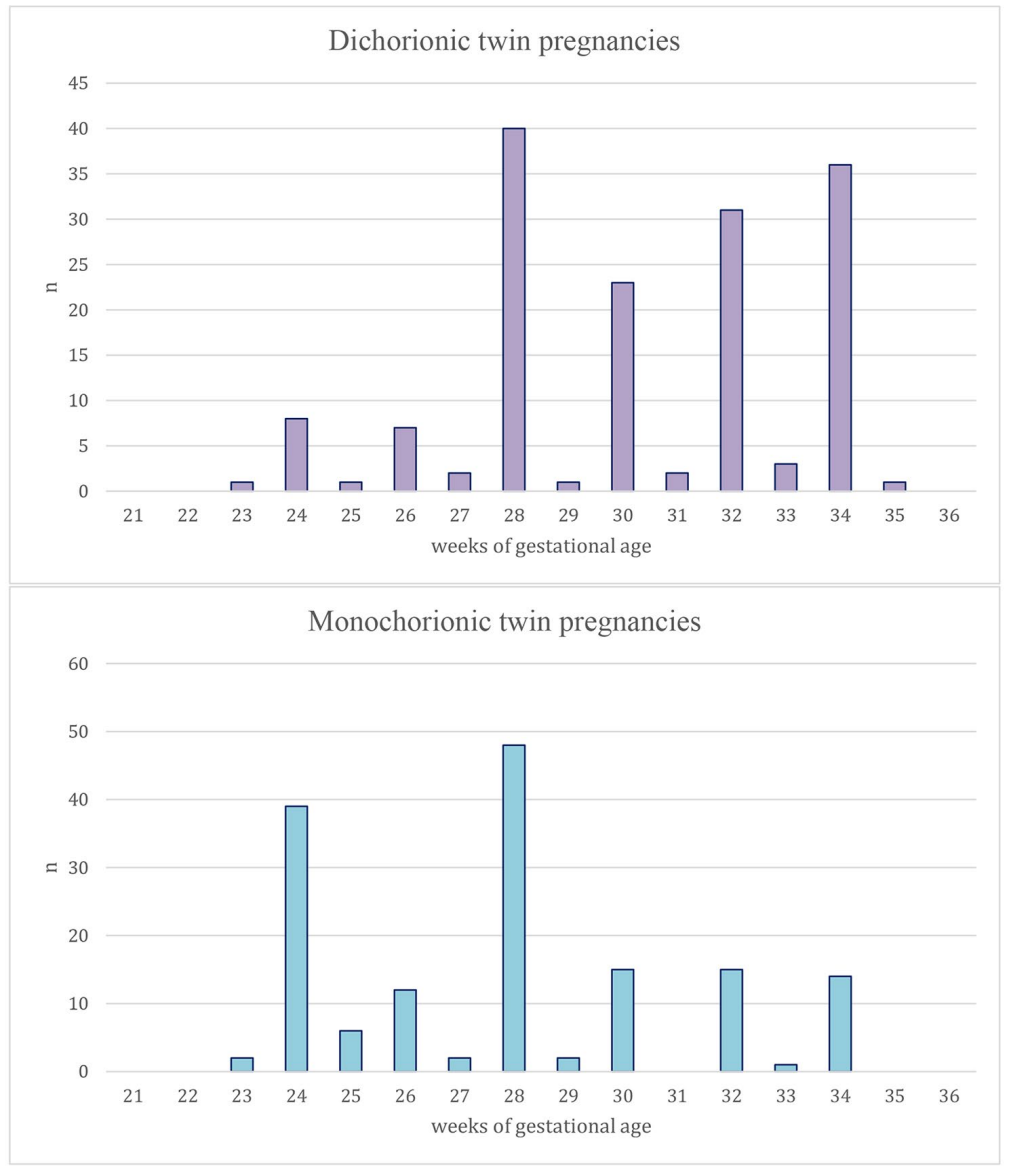
Optimal delivery timing determined by obstetricians and gynecologists as appropriate for impending compromise of selective fetal growth restriction in twin pregnancies

In DC twin pregnancy, more than half (89/156, 57.1%) of the participants answered that they would immediately deliver the twin pregnancy. However, 90.4% (141/156) answered that they would immediately deliver the pregnancy in the same scenario for MC twins. The participants designated 30 weeks (IQR, 23–35 weeks) for DC twins and 28 weeks (IQR, 23–34 weeks) for MC twins as the optimal gestational age of management transition from maintaining pregnancy to immediate delivery (p < 0.001 between DC and MC pregnancies, Fig. 1). That is, in the given situation, the proportion of immediate delivery in MC twin was higher than in DC twin, and that GA was also earlier. This is thought to be not only to prevent IUD of smaller twin, but also to prevent larger co-twin’s IUD and hypoxic brain injury that can come after smaller twin’s IUD simultaneously [4]. Data analyzed by classifying board members among survey participants were added as Supplementary files (Additional files 1 to 4).

### Correlations between the optimal gestational age of management transition and the limit of viability and intact survival

**Fig. 2:**
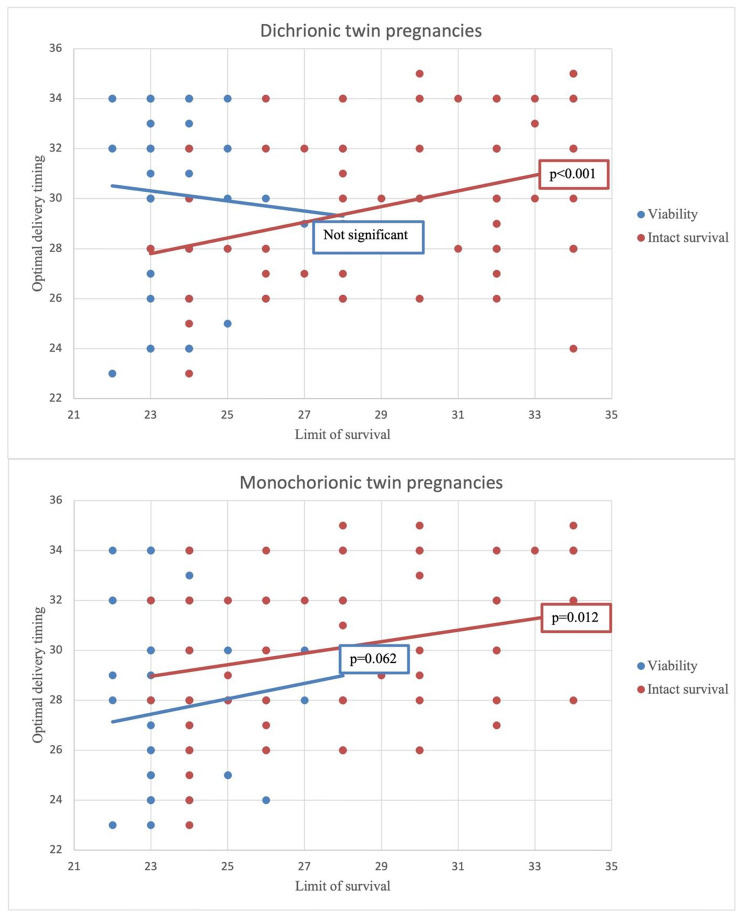
The correlation between optimal delivery timing in impending compromise of selective fetal growth restriction and the limit of survival in neonates

Of the 156 participants, 84.6% participants worked at a hospital with an NICU. The participants regarded 24 weeks (IQR, 22–28 weeks) as the limit of viability and 30 weeks (IQR, 23–35 weeks) as the limit of intact survival in general preterm neonates (Table 4). Figure 2 shows the correlations between the optimal gestational age of management transition and the limit of viability or intact survival in general preterm neonates. The optimal gestational age of management transition in DC twin pregnancy was correlated with the limit of intact survival in general preterm neonates (p < 0.001) but not with the limit of viability (p = 0.211). However, the optimal gestational age of management transition in MC twin pregnancy was associated with both the limit of intact survival (p = 0.012) and viability with marginal significance (p = 0.062). Data analyzed by classifying board members among survey participants were added as Supplementary files (Additional files 3 and 5).

## Discussion

### Principal findings

In the present study, we found that, in situations of twin pregnancies complicated by sFGR with signs of impending IUD of the smaller twin, larger number of participants tended to determine immediate delivery in MC twin pregnancy (DC twin 57.1%; MC twin 90.4%) to prevent IUD of the smaller fetus. The participants tended to decide the delivery timing of twin pregnancies complicated by sFGR as 30 weeks for DC twin and 28 weeks for MC twin pregnancies. While the participants considered the limit of viability as 24 weeks and intact survival as 30 weeks in general preterm neonates, the delivery timing in DC twin pregnancies showed a significant correlation with the limit of intact survival (p < 0.001) but no correlation was observed with viability (p = 0.211). The delivery timing for MC twin pregnancies complicated with sFGR was correlated with both the limit of intact survival (p = 0.012) and viability with marginal significance (p = 0.062).

In situations of impending IUD of twin pregnancies complicated by sFGR, participants tended to decide differently between DC and MC twin pregnancies, specifically whether and when to deliver the twin pregnancy. The management of a growth restricted DC twin fetus is usually similar to the management of a growth restricted singleton [1], with an additional factor of continuing the pregnancy as long as possible in the interests of the appropriately grown co-twin [7]. However, the management of a MC twin pregnancy complicated by sFGR is different from that of singleton or DC twin pregnancies. The purpose of managing these MC twin pregnancies complicated by sFGR is to extend the pregnancy until viability is achieved, while avoiding a single IUD with severe consequences associated with the survival of the co-twin [7]. The difference in the strategy between DC and MC twin pregnancies is attributed to the different fetal circulation system according to chorionicity [7]. In the case of MC twins, two fetuses share a single placenta with vascular anastomosis existing between two fetuses [12]. According to one study, in the case of MC twins, the reported frequency of vascular connections in placentas approaches 95% [13]. This vascular anastomosis may cause sudden and significant fall in vascular resistance of the co-twin at the time of the smaller fetus’s death, resulting in the shunting of blood from the surviving co-twin to the dead one. This leads to hypoperfusion, hypotension, and fetal anemia in the surviving twin, and these phenomena result in tissue hypoxia, acidosis, and tissue damage, particularly in the central nervous system [14].

It can be assumed that the participants tended to decide on immediate delivery at an earlier gestational age not only for smaller twin but also for larger co-twin to prevent IUD or neurologic damage of the co-twin in MC twin pregnancies complicated by sFGR, based on the concerns about these phenomena. Indeed, the optimal gestational age of management transition in a MC twin pregnancy was associated with both the limit of intact survival and viability with marginal significance (p = 0.062), while the optimal gestational age of management transition in a DC twin pregnancy was correlated with the limit of intact survival in general preterm neonates (p < 0.001) but not with the limit of viability. When classifying PTB by GA, it can be divided into moderate-to-later preterm(GA 32–36 weeks), very preterm(GA 28–31 weeks), and extremely preterm(< GA 28 weeks) [15]. Among the participants in this study, there were participants who answered that they would decide to deliver DC twin in given situation on the extremely preterm period, and that would be with the increased risk of adverse neurodevelopmental outcomes. According to a review study, the prevalence of neurodevelopmental impairment increased among survivals as GA increased, that is, very preterm group 5.8%; GA 27 weeks 16.9%; GA 26 weeks 20.2%; GA 25 weeks 32.6%; GA 24 weeks 42.2%; GA 23 weeks 50.3% [16]. Although there may be differences between centers, the participants who chose extremely preterm period for delivery timing of DC twin in given situation, they may look on the opposite side of the number of moderate to severe neurodevelopmental impairment prevalence.

For MC twin, it appears that OBGYN doctors might consider both viability and neurologic outcome important in a MC twin pregnancy and decided to deliver midway between the limit of viability and intact survival. According to one study, regarding the prognosis of the co-twin in the situation of one fetal death in the uterus, the odds of a MC twin death following the IUD of the smaller fetus after 20 weeks of gestation was six times higher compared with that in DC twins, and the risk of neurological abnormality in the surviving MC and DC co-twins were 18% and 1%, respectively [17]. Other complications following a single IUD of the twin, such as preterm delivery (68% vs. 54%), abnormal postnatal cranial imaging findings of the surviving fetus (34% vs. 16%), and neurodevelopmental impairment of the surviving fetus (26% vs. 2%), were relatively higher in a MC twin than in a DC twin [17–19]. Because of this phenomenon, it can be considered that the participants in the survey decided to deliver the MC twin pregnancy complicated by sFGR with signs of impending IUD of the smaller twin at a relatively earlier gestation than a DC twin pregnancy in the same situation, and the intact survival or viability when determining the delivery timing could not be overlooked. However, the participants were more likely to deliver the DC twin pregnancy with sFGR with impending IUD of the smaller twin at the limit of intact survival, because the risk of the co-twin’s death was minimal according to previous reports [1].

According to present study, OBGYNs in South Korea tended to consider viability and intact survival when they determine the delivery timing for impending IUD of smaller fetus in twin pregnancy complicated with sFGR, but the participants might feel difficulties when they face the situation in real world. Besides, discussions with multidisciplinary team, such as neonatologist, and parents will be important. Neonates born prematurely may have problems with viability, intact survival, as well as various complications caused by PTB, such as long term neurodevelopmental disabilities [20], pulmonary dysfunction, and visual dysfunction [21]. Over the decades, the outcome of premature neonates in NICU have improved compared to the past [22], but the risks of adverse outcome comes by preterm birth remain still. According to one study, early preterm birth (GA < 32 weeks) represents about 1.6% of US live births, but they account for more than half (52%) of infant deaths, neurodevelopmental disability and substantial medical complications [23] and some may have fetal origin disease in the future [24]. When it comes to Korean research, recent studies announced that lower GA is associated with elevated risk of adverse neurodevelopmental and respiratory outcome [15]. Furthermore, limit of viability and intact survival is not defined as same gestational age in worldwide, it can differ from country to country [25], and center to center [26, 27]. Therefore, a multidisciplinary approach will help OBGYNs make appropriate decisions that suits individual situation.

According to one survey study, parents of PTB infants tended to save infants at all costs, and they were also prepared for the complications such as disabilities health states [28]. However, these results indicate a tendency and do not represent the opinions of all parents. There may be several variables such as the severity of complications, individual characteristics, such as religion. Assuming that appropriate counseling has been done for the situation, some parents will take a risk from PTB for saving both fetuses, others will want to prolong pregnancy for maturation of larger twin, even if the smaller twin is sacrificed, and some may want intervention. The parents are the one who are responsible for such babies, so their opinions cannot be overlooked. We believe that further studies are needed to evaluate the parents’ perspectives regarding this issue.

Overall, in the dilemmatic situations of impending death of smaller fetus in twin pregnancies complicated with sFGR, OBGYN in South Korea showed tendency to decide on immediate delivery to save a smaller fetus in MC twin pregnancy rather than DC twin pregnancy, and that the GA to decide on immediate delivery was also earlier than DC twin, which was seemed associated with fetal viability along with intact survival.

### Strength and limitations of the study

While there is no guideline on optimal delivery timing for a compromised twin with sFGR, this study analyzed the decision of OBGYN doctors and the correlated factors and characteristics that influence their decisions. As far as we have searched, this is the first study to analyze the tendency of OBGYN doctors to choose immediate delivery or pregnancy prolongation in dilemmatic situations that twin pregnancies complicated with sFGR can experience. In such dilemmatic situations, this study can be helpful and, furthermore, we anticipate that this study could serve as a basis for future guidelines in this area.

However, this study has some limitations. This survey was only distributed in South Korea, so this study and the analysis of data can be a reference for deciding the optimal delivery timing for compromised sFGR twin pregnancies in Korea and targeted at OBGYN doctors regardless of their specialties. Therefore, it would be helpful to establish global guidelines if we could gather more opinions from OBGYN doctors from many countries around the world as well as Korean OBGYN doctors. To this end, it is expected that this study can be helpful when conducting surveys targeting OBGYN doctors in each country or region. Furthermore, in future studies, the survey could be distributed to only maternal-fetal medicine specialists, who specialize in maternal care, as the analysis and comparison between the results among this group of specialists can be helpful for caring and assessing pregnant women who face dilemmatic situation such as cases like these.

## Conclusion

Participants tended to deliver a compromised sFGR twin pregnancy at the gestational age of the limit of intact survival (30 weeks) for DC twin pregnancy and at the midway between the limit of viability and intact survival (28 weeks) for MC twin pregnancy. However, more research is needed to answer this practice pattern and determine actual subsequent neonatal outcomes.

## Electronic supplementary material

Below is the link to the electronic supplementary material.

**Additional file 1: S1.** The perspective of board members among participants on the optimal delivery timing for impending compromise of the smaller twin in selective fetal growth restriction

**Additional file 2: S2.** The perspective of board members among participants on the optimal delivery timing for impending compromise of the smaller twin in selective fetal growth restriction

**Additional file 3: S3.** The perspective of board members among participants on limits of viability or intact survival in preterm neonates

**Additional file 4: S4.** Optimal delivery timing determined by all survey participants and board members as appropriate for impending compromise of selective fetal growth restriction in twin pregnancies

**Additional file 5: S5.** The correlation between optimal delivery timing in impending compromise of selective fetal growth restriction and the limit of survival in neonates by all survey participants and board members

## Data Availability

The datasets used and analyzed during the current study are available from the corresponding author on reasonable request.

## Abbreviations

sFGR: selective fetal growth restriction
IUD: impending intra-uterine death
PTB: preterm birth
OBGYN: obstetricians and gynecologists
DC: dichorionic
MC: monochorionic
KSUOG: the Korean Society of Ultrasound in Obstetrics and Gynecology
NICU: neonatal intensive care unit
