# Supplementary material for: Management of the smaller twin with impending compromise in twin pregnancies complicated by selective fetal growth restriction: a questionnaire-based study of clinical practice patterns
Source: BMC Pregnancy Childbirth. 2023 May 12;23:344. doi: 10.1186/s12884-023-05616-3 (PMC10176903; doi:10.1186/s12884-023-05616-3)
Supplement: Supplementary file 2 — Additional file 2: S2. The perspective of board members among participants on the optimal delivery timing for impending compromise of the smaller twin in selective fetal growth restriction [file 12884_2023_5616_MOESM2_ESM.docx]

**S2. The perspective of board members among participants on the optimal delivery timing for impending compromise of the smaller twin in selective fetal growth restriction.**

|  | **DC** | **MC** | **p-value** |
| --- | --- | --- | --- |
| Optimal delivery timing in term of gestational age at delivery (weeks) | 30 (23–35) | 28 (23–34) | < 0.001 |
| Board members | 30 (23–35) | 28 (23–34) | < 0.001 |

Data are presented as proportion (%) or median (Interquartile range).

Abbreviations: DC, dichorionic; MC, monochorionic
