# Supplementary material for: Management of the smaller twin with impending compromise in twin pregnancies complicated by selective fetal growth restriction: a questionnaire-based study of clinical practice patterns
Source: BMC Pregnancy Childbirth. 2023 May 12;23:344. doi: 10.1186/s12884-023-05616-3 (PMC10176903; doi:10.1186/s12884-023-05616-3)
Supplement: Supplementary file 3 — Additional file 3: S3. The perspective of board members among participants on limits of viability or intact survival in preterm neonates [file 12884_2023_5616_MOESM3_ESM.docx]

**S3. The perspective of board members among participants on limits of viability or intact survival in preterm neonates**

|  | **GA (weeks)** | **EFW (g)** |
| --- | --- | --- |
| Viability | 24 (22–28) | 500 (250–1500) |
| Board members | 24 (22-28) | 500 (250–1000) |
| Intact survival* | 30 (23–35) | 1000 (400–1700) |
| Board members | 28 (23-34) | 1000 (400–1700) |

Data are presented as proportion (%) or median (Interquartile range).

GA, Gestational age; EFW, Estimated fetal weight.

*Intact survival was defined as neonatal survival without neurologic abnormality
