# Supplementary material for: Management of the smaller twin with impending compromise in twin pregnancies complicated by selective fetal growth restriction: a questionnaire-based study of clinical practice patterns
Source: BMC Pregnancy Childbirth. 2023 May 12;23:344. doi: 10.1186/s12884-023-05616-3 (PMC10176903; doi:10.1186/s12884-023-05616-3)

**S4. Optimal delivery timing determined by all survey participants and board members as appropriate for impending compromise of selective fetal growth restriction in twin pregnancies.**

**(a) All survey participants**


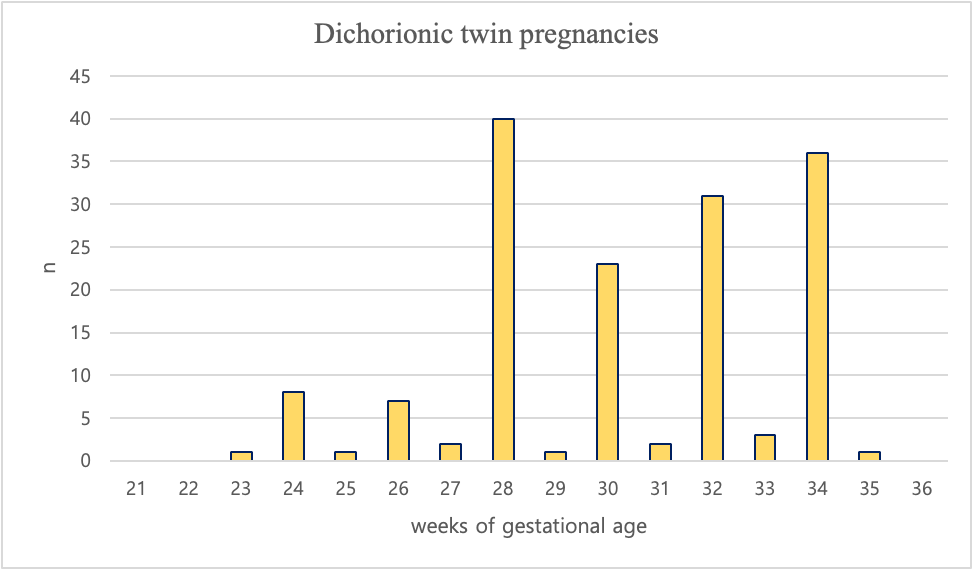

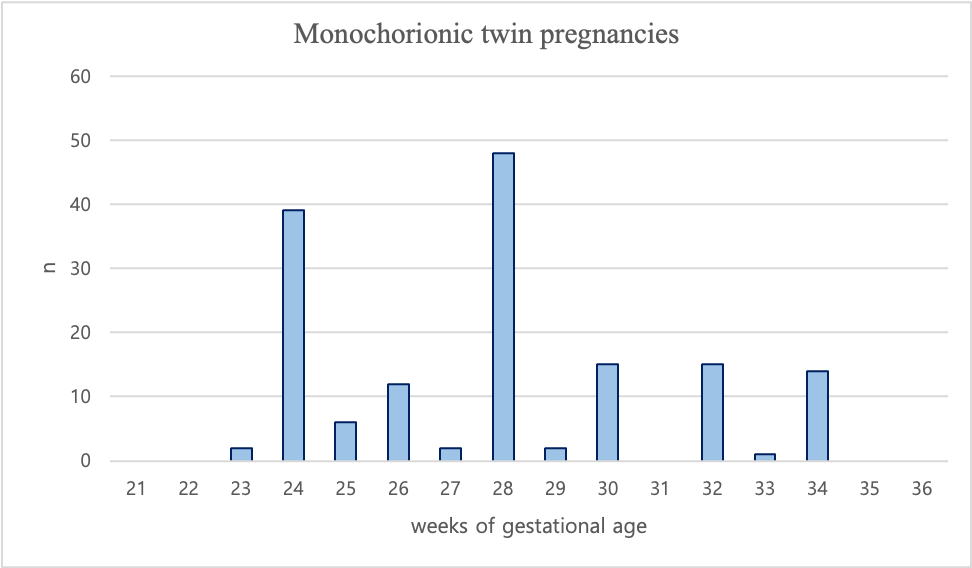


**(b) Only board members**


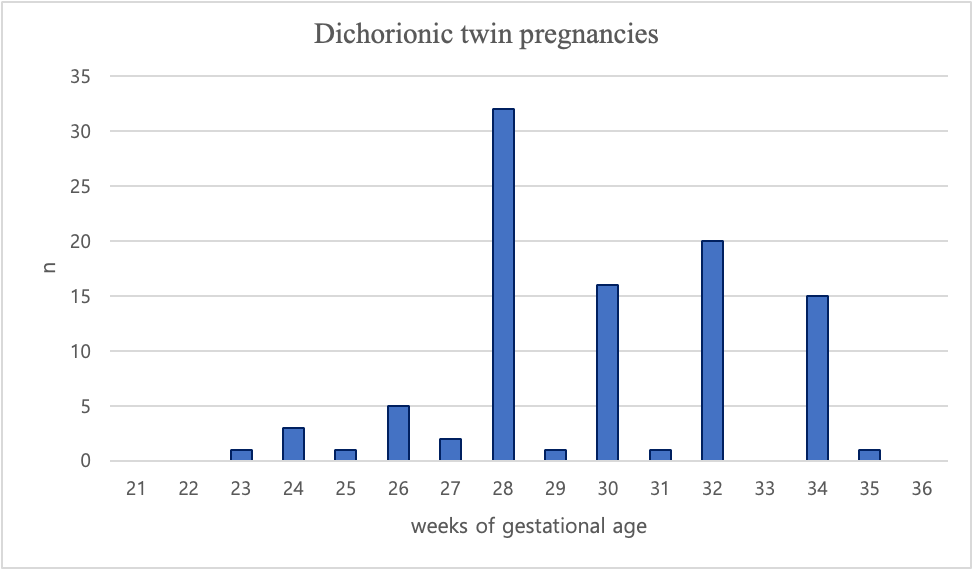

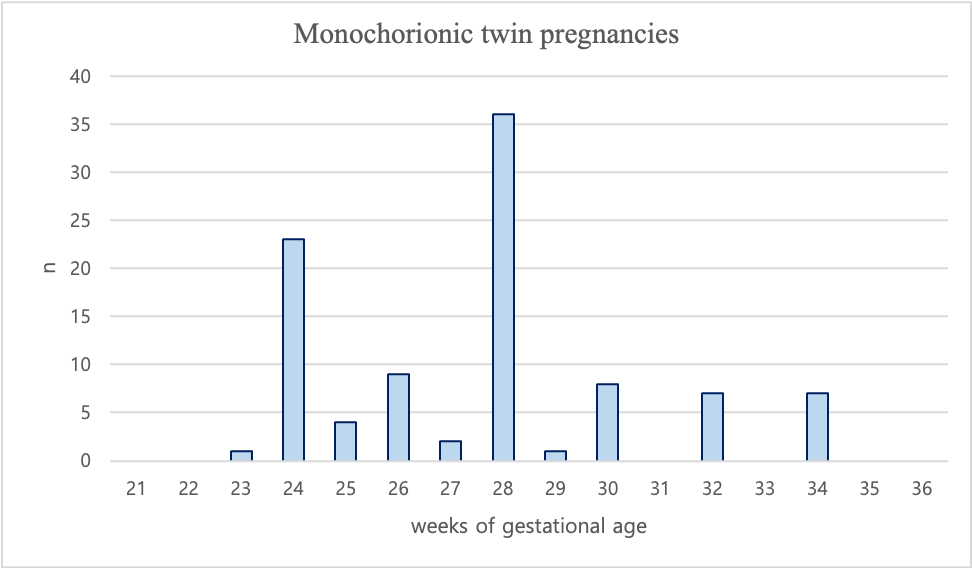

Supplement: Supplementary file 4 — Additional file 4: S4. Optimal delivery timing determined by all survey participants and board members as appropriate for impending compromise of selective fetal growth restriction in twin pregnancies [file 12884_2023_5616_MOESM4_ESM.docx]
