# Supplementary material for: Management of the smaller twin with impending compromise in twin pregnancies complicated by selective fetal growth restriction: a questionnaire-based study of clinical practice patterns
Source: BMC Pregnancy Childbirth. 2023 May 12;23:344. doi: 10.1186/s12884-023-05616-3 (PMC10176903; doi:10.1186/s12884-023-05616-3)
Supplement: Supplementary file 5 — Additional file 5: S5. The correlation between optimal delivery timing in impending compromise of selective fetal growth restriction and the limit of survival in neonates by all survey participants and board members [file 12884_2023_5616_MOESM5_ESM.docx]

**S5. The correlation between optimal delivery timing in impending compromise of selective fetal growth restriction and the limit of survival in neonates by all survey participants and board members.**

**(a) All survey participants**

**
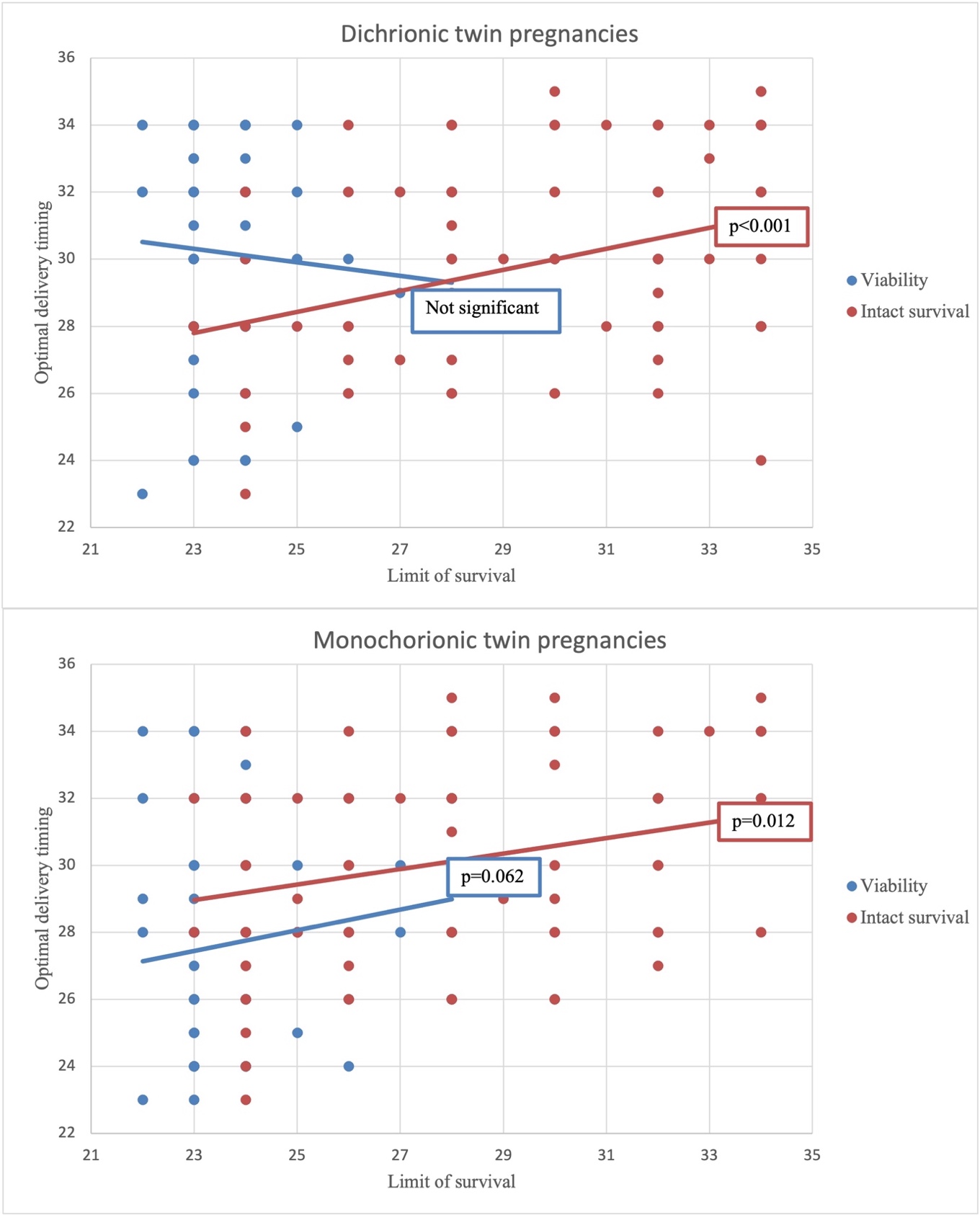
**

**(b) Only board members**


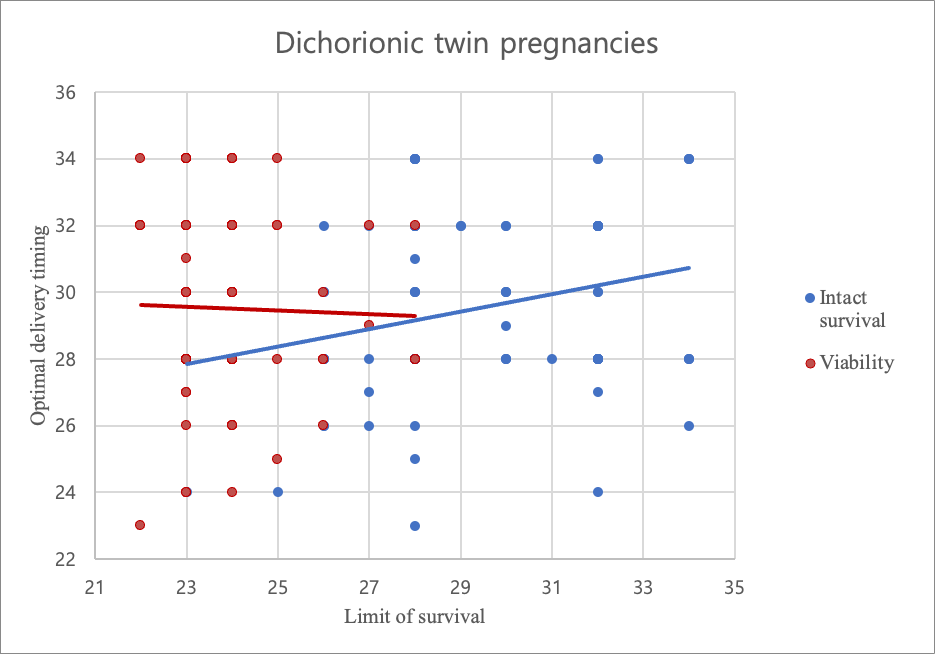

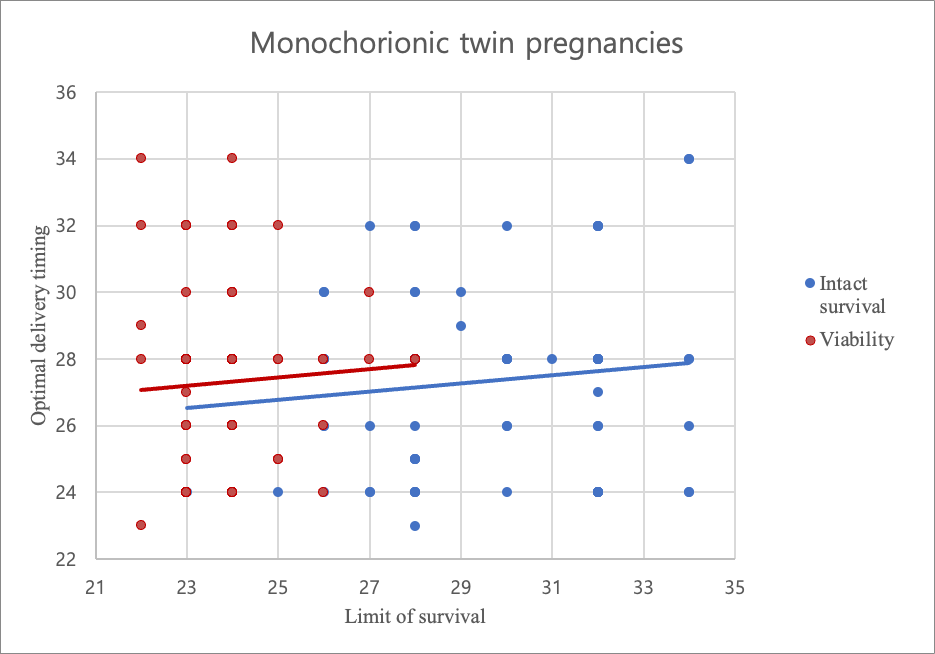


p=0.385

p=0.623

p=0.879

p=0.035
